# Hazard ratio

**DOI:** 10.3325/cmj.2019.60.293

**Published:** 2019-06

**Authors:** Vladimir Trkulja, Pero Hrabač

**Affiliations:** 1 Department of Pharmacology, Zagreb University School of Medicine, Zagreb, Croatia; 2 Department of Medical Statistics, Epidemiology, and Medical Informatics, “Andrija Štampar” School of Public Health, University of Zagreb School of Medicine, Zagreb, Croatia *phrabac@hiim.hr*

We previously depicted the essentials about several measures of association between variables when the *outcome variable* (or *dependent variable*) is binary: (absolute) risk and prevalence difference (simple differences in proportions), and three ratio measures (which quantify relative differences) – odds ratio (OR), risk ratio (RR), and prevalence ratio (PR; computationally the same but conceptually different from RR) (1,2). To exemplify these measures, we outlined hypothetical observational studies aimed to assess an association between a binary *dependent* variable (diagnosis of carcinoma/no diagnosis of carcinoma) and a binary *independent* variable (exposure or no exposure to a certain agent). We pointed out that the ratio measures (OR, RR/PR) could be modeled [ie, log(odds), log(risk/prevalence)]. This allows one to quantify the association between a *continuous* independent variable and a binary dependent variable. In such a case, OR and RR/PR quantify a relative change in odds (OR) or risk/prevalence (RR/PR) of the outcome associated with unit change in the independent variable (eg, the risk of carcinoma if age is 5 years above the mean age of the observed sample/risk of carcinoma at the mean age).

**Figure 1:**
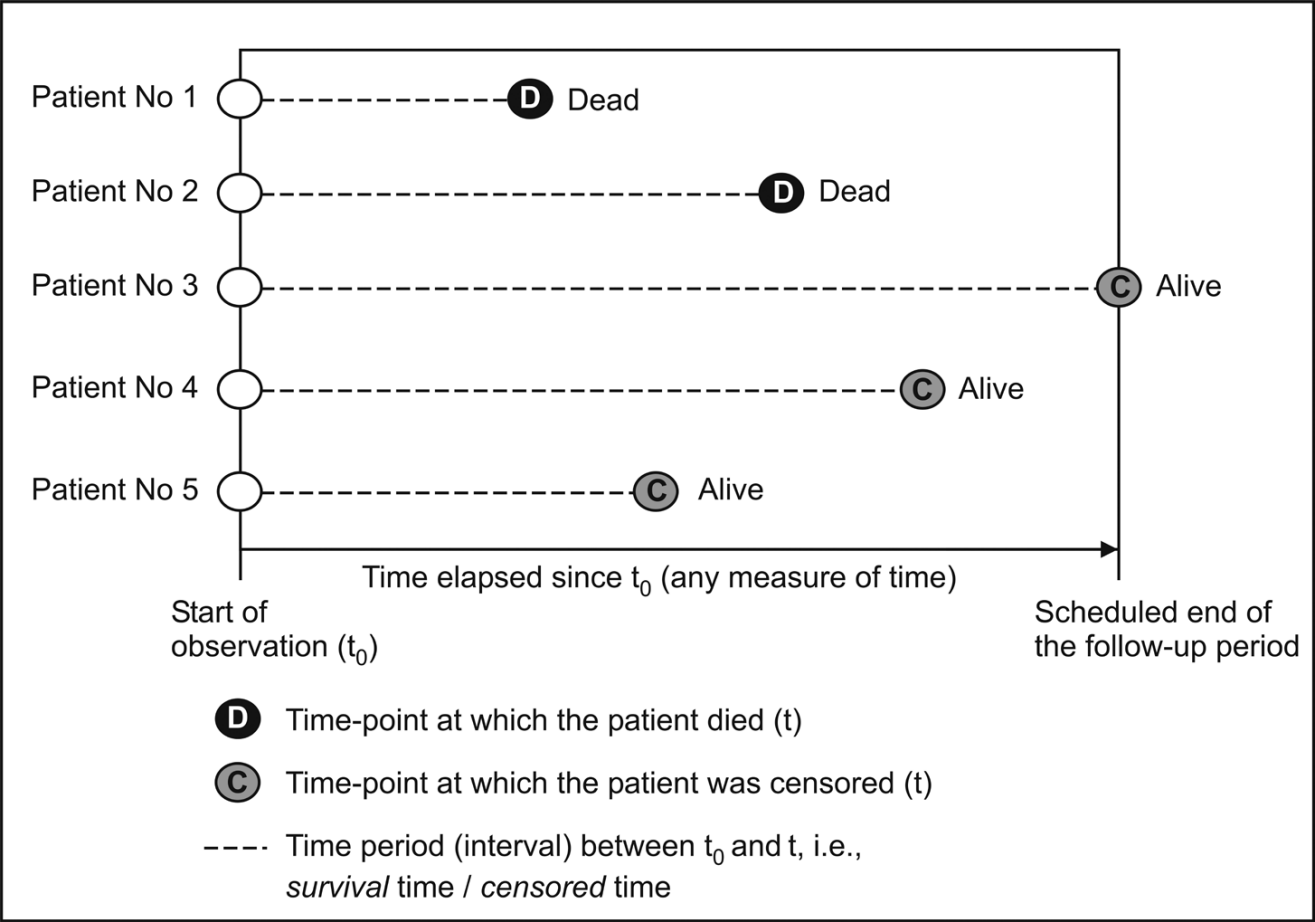
Possible scenarios with patients in a study with time-to-event data (here, event = death).

There are situations, however, when these measures are not adequate for the evaluation of potential associations between presumed independent variables and a binary outcome variable. Let us assume that in a clinical trial patients with advanced carcinoma are randomized to receive a standard treatment or a new treatment. The outcome of interest is death (yes/no). Let us further assume that by the end of the third year since randomization all patients in both groups have died. However, the dynamics of dying (over time), ie, propensity to dying were different: with the standard treatment all the patients have died by the end of the first year, while with the new treatment only around 20%-25% have died by the end of the first year. Clearly, the estimate of an association between the new treatment and the risk of death (ie, estimate of the treatment effect) based on OR or RR would be biased since it would account only for the proportion of patients who died over 3 years and would disregard the important information about the *time* since the start of treatment at which death occurred. One could consider using the *time to death* as an outcome (and compare it between treatments), but these values are typically highly skewed. Moreover, let us assume that in each group there were 5% of patients who received the full treatment but were then lost to follow-up at different times since the start of treatment. *Lost to follow-up* means: “the last time we looked” they were alive, but then for some reason they stopped reporting to the clinic for regular check-ups, so while for the other 95% we know they died, for these 5% we do not know whether they are alive or dead at the end of the third year since the start of treatment. The same would be with patients who survive to the end of the third year – we would know that they survived up to three years, but we would not know their time of death. Under such conditions, how could one compare times to death, when time to death is not known for all the patients? There are two “types” of times in such a scenario: time to death or *survival time*, and time to the end of follow-up (scheduled or early) at which the patient is still alive, the so-called *censored time* (Figure 1). Several methods can be used to analyze such data, ie, to consider the *time* as a dependent variable accounting for the fact that the endpoint is either the *event* or *censoring* (3,4). Initially, the methods were developed to analyze data where the event of interest was indeed death, hence the name *survival analysis*, however, the *event* could be virtually any binary outcome and subject to analysis could be, eg, time to disease occurrence (or recurrence), time to eradication of the disease, time to hospitalization, etc. Therefore, the alternative name for this kind of data are *time-to-event* data. Different methods for time-to-event analysis differently quantify the association between (one or more) independent variable(s) and the dependent variable: they will provide results of formal statistical tests, but also some form of *quantification* of the effects, eg, (estimated or expected) median times to event (which would, for example, illustrate the difference between two treatments) (the so-called non-parametric methods) or estimated ratios of mean times to event (the so-called parametric methods). However, in biomedical research by far the most common effect measure in the analysis of time-to-event is the *hazard ratio* (HR). It arises from a semiparametric regression method of analysis named after its author (Sir David R. Cox) (3,4). In their essence, all survival analysis methods use the observed times (to an event or to censoring) to estimate three functions (3,4): a) the survivor function [*S*(*t*)], which could be defined as the probability (for an individual) to survive (or not experience an event of interest) from the start of observation (t_0_) to and beyond a certain later time (t); b) the hazard function [*h*(*t*)], which could be defined as the probability that a patient experiences the event at time *t*, under the condition that he/she has not experienced it before time *t* (eg, if time is measured in days, that the patient who is alive on day X dies during that day). This function is also referred to as the *hazard rate* or the *instantaneous death rate* (3) (or, correspondingly, an instantaneous probability or risk of an event); c) cumulative hazard function [*H*(*t*)], which could be defined as the cumulative risk of an event by time *t*, ie, an expected number of events that occur in the interval between *t*_0_ and *t*. The *Cox proportional hazard regression* method models log[*h*(*t*)]. It quantifies the association between a categorical (binary, or with more levels) independent variable and the outcome by determining the difference in logarithms of hazard functions between different levels of the independent, eg, for a new vs the standard treatment {log[*h*_N_(*t*)] - log[*h*_S_(*t*)]}; or between a continuous independent variable and the outcome by determining the difference between the log (hazard functions) at two different values of the independent (eg, at age 5 years above the mean age vs at the mean age). The exponent of the difference between these logarithms is the *hazard ratio* (HR). Like some other methods (parametric survival methods), the Cox method assumes that the compared hazard functions are proportional (although, it can be generalized to allow for nonproportional hazards, 4), ie, that their relation over time remains constant (the same).

**Figure 2:**
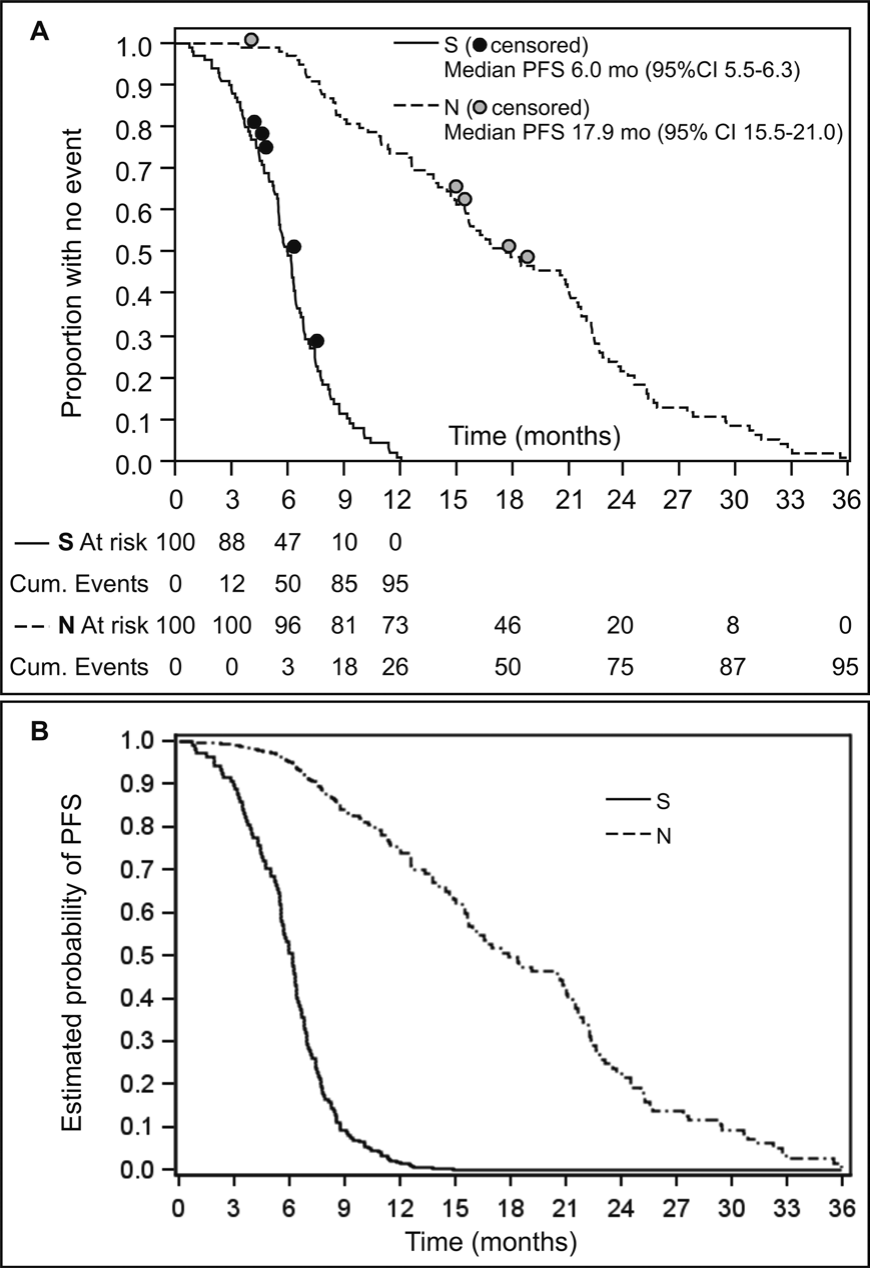
Time-to-event data from the hypothetical (simulated) study depicted in the text: advanced cancer patients are randomized to a standard treatment (S, n = 100) or a new treatment (N, n = 100) and the scheduled follow-up is three years (36 months). The outcome of interest is disease progression or death and the subject of analysis (dependent variable) is time-to-event, ie, *progression-free survival* (PFS). (**A**) Time-to-event data by treatment are summarized by the non-parametric Kaplan-Meier method (KM *survivor curves*). Dots represent censored observations (patients lost to follow-up before the end of the scheduled observation period, see text). Depicted are numbers of patients starting subsequent sub-periods of time who are still without the event (*at risk* patients) and the cumulative number of those experiencing the event. The method estimates median times-to-event, ie, median PFS for S and for N. (**B**) Adjusted curves depicting estimated probability of no event over time (adjusted PFS curves) for S and N obtained by the Cox method (adjustments for age and pathohistological grade).

As a ratio measure, HR quantifies relative hazard, ie, relative risk (RR) and can vary between 0 and infinity: if HR = 1.0, there is no difference between two hazard functions (two risks of the event, each associated with the respective level/value of the independent variable), the numerator one and the denominator one; if HR<1.0, the numerator risk is lower than the denominator; if HR>1.0, the numerator risk is higher. It should be noted: although HR is standardly interpreted as a RR (of an event), the information conveyed by HR differs from that conveyed by the “common” RR determined as a ratio of two proportions of incident patients. HR quantifies *instantaneous relative risk*, while RR quantifies *cumulative risk* –these two estimates may considerably differ for the same data. To illustrate this, Figure 2 summarizes data from the mentioned hypothetical (simulated) clinical trial in which patients with advanced carcinoma were randomized to receive a standard treatment (S, n = 100) or the new treatment (N, n = 100), and are to be followed-up over 3 years after randomization. All patients are >50 years of age and the two groups are similar in this respect (S, mean ± standard deviation [SD] = 72.1 ± 6.2 years; N, mean±SD = 70.1 ± 5.4 years), with a similar proportion of patients with a “worse” pathohistological grade (as opposed to “better”) (S 53%, N 49%). To make the example more realistic, the outcome of interest is *disease progression* or *death*, whichever first, and the dependent variable is *time-to-event* (progression or death), the so-called *progression-free survival* (PFS), a standard dependent variable in oncology studies. In each group, 95% of the patients experienced the event by the end of the third year since randomization and the start of treatment, while 5% in each group were lost to follow-up.

*Cumulative risk* of the event over a period of three years in each group = 95/100 = 95%. A simple cumulative relative risk (N/S) RR = 1.00 (95% CI 0.93-1.08), *P* > 0.999. With adjustment for age and pathohistological grade (to estimate the treatment effect unconfounded by possible effects of these characteristics), relative risk of event for N vs S: (adjusted) RR = 0.99 (95%CI 0.93-1.06), *P* = 0.828. Hence, either way, cumulative RR indicates no difference between treatments regarding disease progression or death over three years, ie, no benefit of the new treatment.

However, the time-to-event (or commonly *survival*) curves clearly indicate that time-to-event (PFS) is considerably different between the two treatments (Figure 2): a simple non-parametric method (compares treatments without adjustments) estimates median time to progression/death (median PFS) for N to be 17.9 months vs 6.0 months for S (*P* < 0.001) (Figure 2A). A parametric method (with adjustment for age and pathohistological grade) estimates mean PFS for S to be 6.7 months (6.1-7.2) and for N to be 20.5 months (18.9-22.2) and gives their ratio (N/S) = 3.07 (95% CI 2.74-3.44), *P* < 0.001 – indicating around three times longer mean PFS (“average” time to disease progression or death) with N, a huge effect of N.

Figure 2B shows *adjusted* (for age and pathohistological grade) curves of PFS for S and N (ie, of the estimated probability of no progression/death) over time by the Cox method. The (adjusted) hazard ratio, HR = 0.072 (95% 0.042-0.115), *P* < 0.001, indicates a great reduction (by around 92.8%) in the *instantaneous risk* of the event with N: whatever the risk of event with the S treatment for any subsequent section of time, the risk with N is 92.8% relatively lower. This is illustrated by the estimated proportion of patients still without the event at any time point since t_0_: for example, at three months in the S group, the estimated proportion of patients without an event is 88.0%. With N the estimate is 99.1%, and could be obtained as

(Proportion without event with S)^HR^ = 0.88^0.072^ = 0.991.

In absolute terms, this is a difference in the probability of a three-month PFS of 11.1% (91.1-88.0). The probability of surviving without disease progression to any (and beyond) subsequent time point would always be higher with N for the same relative amount, but the absolute amount would change in line with the changes of the PFS with the S treatment. For example, the probability of PFS at 10 months is 6.05% with the S treatment and 81.7% with N [100 × (0.0605^0.072^)] – absolute difference of 75.65%.

Benefits of the new treatment might be illustrated also by the fact that the (adjusted) estimated time needed for 50% of patients to experience the event (median PFS) is close to 6 months with S and close to 18 months with N (virtually the same as by the non-parametric method). In oncology, such differences would be considered a huge and a greatly important effect of any new potential treatment for advanced stages of any malignant disease.
